# Study on the Cognitive Characteristics Induced by Changes in the Intensity, Frequency and Duration of Vibratory Stimuli

**DOI:** 10.3390/bs13050350

**Published:** 2023-04-22

**Authors:** Mi-Hyun Choi, Kyu-Beom Kim, Ye-Jin Kim, Ji-Su Kim, Hyung-Sik Kim, Soon-Cheol Chung

**Affiliations:** 1 Biomedical Engineering, Research Institute of Biomedical Engineering, School of ICT Convergence Engineering, College of Science & Technology, Konkuk University, Chungju 27478, Republic of Korea; kwjcc486@kku.ac.kr (M.-H.C.);; 2 Department of Mechatronics Engineering, School of ICT Convergence Engineering, College of Science & Technology, Konkuk University, Chungju 27478, Republic of Korea

**Keywords:** vibration, frequency, intensity, stimulation duration, cognitive characteristics

## Abstract

The purpose of this study is to analyze the cognitive characteristics that can be induced by vibration stimuli at two intensities, three frequencies, and five presentation periods. The experiment was conducted on 20 right-handed adult males, and a subjective evaluation was performed using a questionnaire. Regression analysis was performed to observe the parameters affecting cognitive characteristics according to changes in intensity, frequency, and stimulation duration. The regression analysis results showed that the cognitive characteristics affected by changes in intensity, frequency, and stimulation duration were “heavy”, “bold”, “thick”, and “light”. The cognitive characteristics affected by two-variable combinations were “deep”, “clear”, “vibrating”, “dense”, “numb”, “blunt”, “shallow”, “fuzzy”, and “soft”. Cognitive characteristics affected by either intensity, frequency, or stimulation duration were “fast”, “pungent”, “skinny”, “thin”, “slow”, “ticklish”, “tingling”, “prickling”, “tap”, and “rugged”. By observing the cognitive characteristics that can be induced by the combination of intensity, frequency, and stimulation duration, we confirmed that in addition to intensity and frequency, the stimulation duration is an important factor that influences the induction of various cognitive characteristics. The results presented in the study can be used to enhance the utility of haptic surfaces for extended reality applications.

## 1. Introduction

The tactile sensations induced by a vibratory stimulus are reportedly influenced by the stimulus frequency, intensity, contact area, stimulation duration, and stimulation pattern [1,2,3,4,5,6]. The majority of the studies on this topic focus on the frequency and intensity of a vibrating stimulus [2,4,6,7] because they have a significant effect on the user’s perception of touch. In our previous study, we investigated vibration stimuli at various frequencies and intensities and analyzed and reported the cognitive characteristics evoked by the stimuli [8,9,10].

However, a study on the cognitive characteristics that can appear at a combination of variables other than frequency and intensity has not yet been conducted. One of the additional variables that our research team is focusing on is the stimulus presentation time. Guemann [11] applied vibration stimuli to the upper arm at different stimulus presentation times (60, 100, and 140 ms) and reported that as the stimulus duration increased, the vibration became stronger. He also reported that the recognition rate appeared differently according to the stimulus presentation time. Bochereau [12] presented vibration stimuli at different stimuli presentation times (100, 200, 300, 500, 600, and 700 ms) and intensities and reported that the stimulus duration and intensity had an inverse relationship. Although previous studies confirmed that the difference in perceived intensity varies according to the presentation time of the vibration stimulus, to our knowledge, no previous study has reported how cognitive characteristics change according to the stimulus presentation time. Furthermore, we have consistently investigated vibrotactile stimuli and found that an excessive stimulus presentation time may induce adaptation in the user or even pain in some cases. Conversely, it has been empirically shown that if the stimulus presentation time is too short, the user may not feel the tactile sensation.

To deliver more diverse and realistic tactile information in the use of a haptic interface, a quantitative study on the cognitive characteristics that can be induced by changes in stimulation duration, intensity, and frequency, is needed. Further, securing the basic data on two or more variables (instead of one) that change at the same time and the type of cognitive characteristics they induce will not only contribute to the existing literature but also enhance the utility of a haptic interface.

Our previous studies confirmed that various cognitive characteristics were distinguished by the combination of various vibration frequencies and intensities [8,9,10]. Moreover, it was also found, based on previous studies, that the intensity varied when the stimulus presentation time of the vibration stimuli was different [11,12]. Based on these findings, this study was conducted based on the assumption that if the stimulus presentation time, which is one of the variables capable of presenting vibration stimuli, is varied, and the frequency and intensity of stimuli are presented, the cognitive characteristics may be changed according to the differences in the degree of changes of individual variables, such as vibration frequency, intensity, stimulus presentation time, and also according to the combination of frequency, intensity, and stimulus presentation time.

In this study, we observed the changes in various cognitive characteristics according to the stimulation duration and (especially) the vibration intensity and frequency. We attempted to determine the relationship between the variables and cognitive characteristics. Based on the two intensities and three frequencies analyzed in our previous study, stimuli were applied to the distal interphalangeal joint of the right index finger at five durations. We aimed to observe the various cognitive characteristics that can be induced based on a combination of intensity, frequency, and stimulation duration.

## 2. Materials and Methods

### 2.1. Preliminary Experiment Conducted to Select Stimulation Durations

Based on the results of our previous research [10], among the three intensities (1st stage: 0.25 G, 2nd stage: 0.38 G, 3rd stage: 1.3 G) and eight vibration frequencies (10, 50, 100, 150, 200, 225, 250, 300 Hz), the two intensities (low intensity: 0.25 G, high intensity: 1.3 G) and three frequencies (100 Hz, 225 Hz, 300 Hz) that produced distinct differences in cognitive characteristics were first selected.

To select the range of stimulation durations, which is the main parameter analyzed in this experiment, a preliminary experiment was conducted on eleven adult males (23.4 ± 1.3 years).

Regarding the stimulation duration of 100 ms used in our previous study [8,9,10], vibration stimuli of two intensities and three frequencies were selected before their application on the distal interphalangeal joint of a subject’s right index finger for seven different durations (10 ms, 100 ms, 1000 ms, 2000 ms, 3000 ms, 4000 ms, 5000 ms). The presence or absence of pain and the time for which the presented stimulus was felt were checked for each stimulation duration. The residual time of a stimulus was more than 15 s when the stimulus was presented for 4000 ms and 5000 ms, and the subject felt frequent pain. The pain was evoked in two subjects when the stimulation time was 4000 ms at a high intensity (1.3 G) and a frequency of 300 Hz. Furthermore, pain appeared in four subjects at a stimulation time of 5000 ms at the same intensity and frequency. Therefore, to exclude pain and residual effects as much as possible, the following five stimulation durations were selected for the experiment: 10 ms, 100 ms, 1000 ms, 2000 ms, and 3000 ms. With these stimulation times, the residual time of the stimulus was less than approximately 10 s.

### 2.2. Preliminary Experiment Conducted to Create a Subjective Evaluation Questionnaire

A subjective evaluation questionnaire was prepared to analyze the changes in cognitive characteristics according to intensity, frequency, and stimulation duration. Responses related to tactile, temperature, pain, and kinesthetic senses were collected based on the subjective evaluation questionnaire used in our previous study [8,9,10]. In addition, the meaning of each word was examined using the standard Korean dictionary published by the National Institute of the Korean Language (https://stdict.korean.go.kr/main/main.do (accessed on 19 April 2023)). After removing words with overlapping meanings, 46 words were collected, and a preliminary experiment was conducted to create a subjective evaluation questionnaire.

Vibration stimuli with different intensities, frequencies, and stimulus presentation times were randomly applied to the distal interphalangeal joint of the index finger of the right hand in 11 males (23.4 ± 1.3 years) once at a time. All the words describing the senses which were felt were collected. The words used in the subjective evaluation experiment were selected based on the words expressed by more than 20% of the subjects. The 24 selected words were “skinny”, “light”, “ticklish”, “bold”, “deep”, “slow”, “thick”, “heavy”, “stubby”, “soft”, “vibrating”, “clear”, “fast”, “dense”, “acrid”, “pungent”, “thin”, “shallow”, “prickling”, “rugged”, “numb”, “tingling”, “tap”, and “fuzzy”. Each word was assessed on a 5-point scale (1 point: no feeling, 5 points: strong feeling) to mark the subjective evaluation questionnaire.

To evaluate the reliability of the fabricated questionnaire, Cronbach’s alpha value, which can indicate the internal consistency of the subjective evaluation questionnaire, was confirmed. Cronbach’s alpha value was performed through reliability analysis of PASW Statics 26. The Cronbach’s alpha value of the produced subjective evaluation score was 0.636, and when individual items were deleted, the Cronbach’s alpha value also showed no significant change (range of Cronbach’s alpha values: 0.606 to 0.655).

### 2.3. Vibration Stimulation System

Various vibration frequencies, intensities, and stimulus presentation times were applied using a self-developed vibration stimulation system [13]. The developed system consisted of a control unit, a drive unit, and an actuator, and it can be operated with a PC or manually. The control unit controls the stimulation frequency, the stimulation intensity, and the stimulation time. The drive unit amplifies the stimulus signal to drive the coil that is the actuator. The solenoid coil, the actuator, was fixed on a permanent magnet. The vibration occurs according to the magnitude and frequency of the current flowing in the coil according to the electromagnetic induction law. The finger to present the stimulus was placed on the coil so that mechanical tactile stimulation could be presented in the form of vibration. The main processor for controlling stimulation variables was the AVR-type ATMEGA128 processor (Microchip Technology Inc., Chandler, AZ, USA). The 12-bit digital-to-analog converter (DAC), AD7545A (Analog Device, Wilmington, MA, USA), was used to generate stimulation signals in the form of sinusoidal waves and to vary the stimulation frequency. The stimulation frequency may be changed to 80 steps by 5 Hz in a range of 0 to 400 Hz. Using Digital potentiometer AD5290 (Analog Device, Wilmington, MA, USA), the sinusoidal signal output from the DAC was divided into 256 steps to control the stimulus intensity. The start and end of the stimulus could be controlled from tens of microseconds to tens of seconds by controlling the digital value input to the DAC. The drive unit used a 20 W audio power amplifier LM1875 (National Semiconductor, Santa Clara, CA, USA) to supply sinusoidal signals from the control unit to the coil. A solenoid coil was simply configured by winding copper wire with a diameter of 0.3 mm 200 times on a cylindrical frame with a diameter of 10 mm printed with a 3-D printer. The operation of the tactile stimulator was confirmed using the MMA7260Q (Freescale Semiconductor, Austin Texas, TX, USA) 3-axis accelerometer. An accelerometer was attached to the solenoid coil in contact with the finger, and the behavior of the stimulus frequency, stimulus intensity, and stimulus time according to the control signal was checked using the digital oscilloscope TDA3044B (Tektronix, Beaverton, OR, USA). Stimulation parameters were controlled using the software E-Prime (Psychology Software Tools, Inc., Sharpsburg, PA, USA) installed on a PC. The area of the vibration stimulus applied to the fingers was 1.8 × 1.8 cm^2^.

### 2.4. Experiments

Experiments were conducted on 20 right-handed adult males (24.4 ± 1.7 years) who had a normal cognitive function. This study was approved by the Institutional Bioethics Committee of Konkuk University (IRB project number: 7001355-202011-HR-408) and complied with the regulations of the Helsinki Declaration. Before the experiment, participants were informed about the study and provided written informed consent. Before the experiment, a sufficient explanation was given to all subjects about the definition of the terms of cognitive characteristics so that they could accurately express the cognitive characteristics of the tactile stimuli they felt.

**Figure 1:**
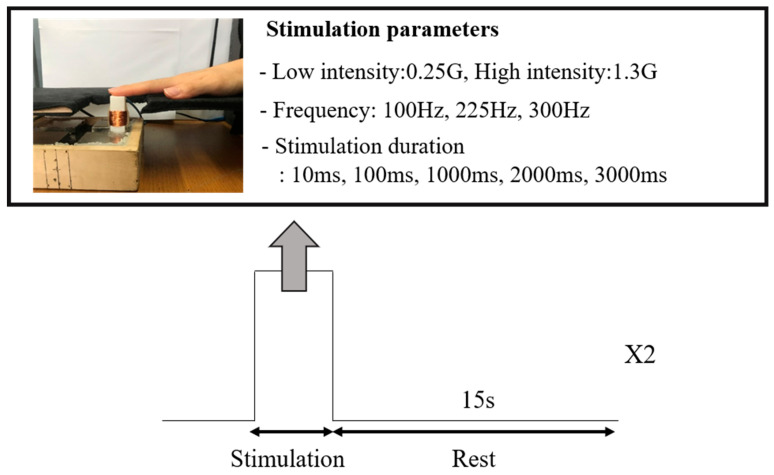
Experimental Design.

One trial of the experiment consisted of a stimulation phase (10 ms, 100 ms, 1000 ms, 2000 ms, 3000 ms) during which a stimulus was applied and a rest phase (15 s), during which no stimuli were applied (Figure 1). Thirty stimulation parameters were used based on the combinations of two intensities, three frequencies, and five stimulation times selected in the preliminary experiment; one of these 30 parameters was randomly selected for a trial. The stimulus under each parameter was applied twice, and a subjective evaluation was performed. To prevent the subject from adapting to the stimulus, the interval between the applications of two different types of stimuli was 5 min. Additionally, the experiment was divided into two days, considering the physical condition of the subjects, because the long experiment time could cause fatigue to the subjects, adaptation to tactile stimuli, and a decrease in concentration.

Regression analysis (PASW Statics 26) was performed to observe the parameters that affect the cognitive characteristics according to changes in intensity, frequency, and stimulation duration.

## 3. Results

The results of the regression analysis showed that the cognitive characteristics affected by the changes in intensity, frequency, and stimulation duration were “heavy”, “bold”, “thick”, and “light”. The regression formula obtained for each cognitive characteristic is shown in Table 1. Evidently, “heavy”, “bold”, and “thick” were positively correlated with intensity and stimulation durations and were negatively correlated with frequency. In contrast, “light” exhibited a negative correlation with intensity and stimulation duration and a positive correlation with frequency.

Table 1 also lists the cognitive characteristics that were affected by any two variables among intensity, frequency, and stimulation duration. Cognitive characteristics that exhibited a positive correlation with intensity and stimulation duration were “deep”, “clear”, “vibrating”, “dense”, and “numb”, and the cognitive characteristics that exhibited a negative correlation with intensity and stimulation duration were “shallow” and “fuzzy”. “Stubby” showed a positive correlation with intensity and a negative correlation with frequency. Further, “soft” exhibited a negative correlation with intensity and a positive correlation with stimulation duration.

Finally, Table 1 lists the cognitive characteristics that were affected by one of the studied variables. The cognitive characteristics that had a positive correlation with intensity were “fast” and “pungent”, whereas “skinny” and “thin” exhibited negative correlations with intensity. “Slow”, “ticklish”, “tingling”, and “prickling” exhibited a positive correlation with stimulation duration, and “tap” showed a negative correlation with stimulation duration. Furthermore, “rugged” exhibited a negative correlation with frequency.

The polarities (positive or negative) of the variables (intensity, frequency, stimulation duration) affecting each cognitive characteristic are summarized in Table 2.

## 4. Discussion

In this study, the three variables of vibration frequency, intensity, and stimulus presentation time were applied to the first knuckles of the fingers of the right hand. The results on the cognitive characteristics that could be induced by the combination of the three variables were presented using subjective scores. The cognitive characteristics that could appear by the combination of the three variables were “heavy”, “bold”, “thick”, and “light”. The cognitive characteristics that could be presented by the combination of intensity and time were “deep”, “clear”, “vibrating”, “dense”, “numb”, “shallow”, “fuzzy”, and “soft”. The cognitive characteristic that could appear by the combination of intensity and frequency was “blunt”. The cognitive characteristics that were affected only by intensity were “fast”, “pungent”, “skinny”, and “thin”. The cognitive characteristics that could be presented by adjusting the stimulus presentation time were “slow”, “ticklish”, “tingling”, “prickling”, and “tap”. Furthermore, the cognitive characteristic that could be presented only by frequency changes was “rugged”.

In our previous studies [8,9,10], we conducted step-by-step research to extract the changes in cognitive characteristics according to various parameters of vibration stimulation and presented the results. Furthermore, Chun et al. [14] tested stimuli at various vibration frequencies (25, 40, 50, 75, 85, 105, 120, 170, 220, 260, 290 Hz) and intensities (0.20, 0.45, 0.60, 0.80 V). They classified the words defining the stimuli based on whether they were easy to use or not. Words related to homogeneity (irregular, regular, etc.), height, depth (deep, low, shallow, etc.), stimulus range (bold, skinny, etc.), and rigidity (hard, curved, etc.) were not appropriate for characterizing vibratory stimuli. In contrast, words related to strength (strong, weak), degree of shaking (shaken, soft), thickness (bold, thick, skinny, thin), weight (heavy, light), and spacing (sparse, dense) were reportedly suitable for expressing the tactility of a simple vibration stimulus. Kim et al. [15] and Kyung et al. [16] reported that as the intensity of a vibration stimulation in the high-frequency band increased, the subjects experienced the surfaces more as coarse, rough, and less soft. Hwang and Hwang [17] reported that the human sensibility for fingertip vibrations might vary depending on the direction and frequency of vibration (20, 40, 80, 160, 320 Hz). Emotions corresponding to “light”, “sensitive”, “repulsive”, “ticklish”, and “exciting” could be evoked. However, positive emotions such as “satisfying”, “enjoyable”, “likable”, and “pleasing” were difficult to induce through fingertip vibration.

In this study, by investigating the effects of stimulation duration in addition to frequency and intensity and based on the results of previous studies that observed tactile cognitive characteristics mainly according to frequency and intensity, we intended to analyze cognitive characteristics based on a regression equation. Cognitive characteristics expressed based on a combination of intensity (low intensity, high intensity), frequency (100, 225, 300 Hz), and stimulation duration (10 ms, 100 ms, 1000 ms, 2000 ms, 3000 ms) changes are summarized as follows.

First, among the subjective evaluation vocabularies presented in this study, the word pairs specified as antonyms in the standard Korean dictionary are “heavy”–“light”, “deep”–“shallow”, “bold”, “skinny”, “thick”–“thin”, and “fast”–“slow”. “Heavy” appeared as the stimulation duration increased at the high intensity and as the frequency decreased. The cognitive characteristic “light” was induced as the stimulation duration decreased at low intensities and as the frequency increased. “Deep” was expressed as the stimulation duration increased at the high intensity, and “shallow” appeared when the stimulus presentation time decreased under the low intensity. “Heavy”–“light” (heaviness) and “deep”–“shallow” (spatiality) were cognitive characteristic pairs for which the stimulus variables that evoked one response (frequency, intensity, or stimulus presentation time) were the opposite of those which evoked the other.

In a previous study [10], “heavy”–“light” was reported to be a contradictory cognitive characteristic pair that appeared according to whether the intensity was high or low at 100 Hz and 225 Hz. In this study, the word pair “heavy”–“light” appeared when the patterns of frequency and stimulation duration, in addition to that of intensity, were opposite. Chun et al. [11] reported that the words “deep” and “shallow”, which indicate depth, were not suitable to express a vibration stimulation; however, the study produced limited results because the stimulation targeted only the frequency changes. Choi et al. [10] showed that the cognitive characteristic score for ”shallow” increased as the intensity decreased at a frequency of 225 Hz. Similar to the results of previous studies, in this study, the appearance of “shallow” was found to be unrelated to the changes in frequency but related to the stimulation duration and intensity. In addition, the characteristic pair “deep”–“shallow” could be formed by controlling the intensity and duration of stimulation.

The words “Bold” and “thick” used to respectively express the shape and thickness of an object, appeared as the stimulation duration increased and the frequency decreased at high intensities. The antonyms “skinny” and “thin” were found to appear only at low intensities. A previous study [10] reported that the cognitive characteristic of “thick” appeared predominantly as the intensity increased in the high-frequency band of 100–225 Hz; however, the results on “skinny” and “thin” were not reported. “Fast–slow”, a cognitive characteristic pair related to stimulus speed and temporality, did not appear when the opposite pattern of stimulus variables was used. In previous studies, “fast” appeared as the intensity increased only at 300 Hz. In this study, “fast” appeared at high intensities, and the antonym “slow” appeared as the stimulation duration was increased.

Among the cognitive characteristics that express visual evaluation, “clear” and “fuzzy” can be considered antonyms in terms of their meaning; however, they are not defined as antonyms in the Standard Korean Dictionary. “Clear” appeared as the stimulation duration increased at the high intensity, whereas “fuzzy” was a cognitive characteristic that was likely evoked as the stimulation duration decreased at low intensities. Although the cognitive characteristics “clear” and “fuzzy” are not antonyms, they can be evoked using the converse combinations of intensity and stimulation duration.

Among the words suggested for subjective evaluation are “prickling” and “pungent”, and the kinesthetic words “tingling” and “numbness” express the sensation of pain. The cognitive characteristics “prickling” and “tingling” were induced as the stimulation duration increased, and “numbness” was induced as the stimulation duration increased at high intensities. “Pungent” appeared only at high intensities. The cognitive characteristics related to nociception and myesthesia could be induced by increasing the intensity and stimulation duration and were not significantly related to frequency changes.

In previous studies, as the intensity increased at 225 Hz and 250 Hz, the intrinsic property of vibrational stimulation, “vibrating”, became dominant [10]. In this study, the cognitive trait was induced not only by high intensities but also by increasing the stimulation duration. Among the words expressing tactility, “dense”, a cognitive characteristic that can express gaps was also found to be induced by increasing the stimulation duration at high intensities. The word expressing surface tactility, “soft”, was generated when the stimulation duration was increased at low intensities. “Soft” was a cognitive characteristic that did not appear when the variables were frequency and intensity, as was the case in our previous studies. In this study, we found that “soft” could only be induced by the combination of stimulation duration and intensity.

Choi et al. [8,9] reported that the cognitive characteristic of “ticklish” appeared predominantly at high frequencies in the range of 200–300 Hz. In this study, the word “ticklish” was found to be uncorrelated to frequency; however, when combined with the results of previous studies, we found that “ticklish” could be induced by increasing the stimulation duration with a high-frequency vibration stimulus. “Tap”, the dynamic cognitive characteristic most frequently experienced by most subjects, showed significant changes only with the stimulation duration and appeared only at shorter stimulation durations.

The cognitive characteristic “stubby”, which represents the sense of a surface, was expressed when the frequency was decreased to 10, 100, and 250 Hz at high intensities, similar to the results of this study and previous studies [10]. Further, the cognitive characteristic “rough” was negatively correlated with frequency and was expressed as the frequency decreased.

Previous studies reported only the results of intensity as a function of frequency, cognitive characteristics according to frequency and intensity changes, or the suitability of the words for the expression of the stimulus. Reports on the cognitive characteristics that appear at a combination of variables other than frequency and intensity are relatively scarce. In this study, the cognitive characteristics that could be induced by a combination of three variables, namely, frequency, intensity, and stimulation duration, were analyzed. In addition to vibration frequency and intensity, stimulation duration was also found to be an important factor that influences the induction of various cognitive characteristics. The results of this study also showed that a broader range of tactile sensations could be presented with the combination of the three variables. The various cognitive characteristics that can be evoked through the combination of frequency, intensity, and stimulation duration can be used to improve the usage of haptic interfaces. Therefore, for all extended reality platforms using haptic interfaces (virtual, augmented, mixed, and substitutional reality), the results of this study may be utilized to match parameters that allow users to experience various and realistic tactile sensations. In social applications, the use of vibration stimuli can enhance human communication by providing an additional sensory channel. Additionally, in the field of entertainment, the results could be used to create more immersive and realistic experiences in gaming and virtual reality. In technological applications, the research results could be applied to the design of human-machine interfaces. For instance, vibration stimuli could be used to provide haptic feedback in touchscreens, which can improve the usability and accessibility of the interface. In medical applications, vibration stimuli could be used in rehabilitation programs to enhance motor learning and improve sensory-motor integration in patients with neurological disorders. However, further research is needed to explore the full potential of vibration stimuli and their applications in different contexts.

Firstly, the current study was conducted on a sample of right-handed adult males, which may limit the generalizability of the results. Future studies could replicate the findings on a more diverse sample, including females and left-handed individuals, to determine if the effects of vibration stimuli are consistent across different populations. Secondly, the current study focused on subjective evaluations of cognitive characteristics using a questionnaire. Future studies could use objective measures, such as physiological measures, to validate the subjective reports of cognitive characteristics induced by vibration stimuli. Thirdly, As noted in previous research, the perception of vibration stimuli can be influenced by factors such as the location and type of the body part stimulated, the presence of other sensory cues, and the task demands placed on the user. Future research should explore how these cognitive characteristics might vary in different contexts and with different types of tasks. Finally, the current study investigated the effects of vibration stimuli on cognitive characteristics using only three frequencies. Future studies could investigate a wider range of frequencies to determine the optimal frequency for inducing specific cognitive characteristics.

## Figures and Tables

**Table 1:** Results of Regression Analysis of Cognitive Characteristic Scores for Three Variables: Intensity, Frequency, and Stimulation Duration.

Variable Combinations	Cognitive Characteristics	Regression Formula
Three variables	Heavy	y = 0.761 + 0.56 × intensity − 0.248 × frequency + 0.106 × duration (intensity: *p* < 0.001, frequency: *p* < 0.001, duration: *p* = 0.001)
	Bold	y = 0.664 + 0.540 × intensity − 0.12 × frequency + 0.064 × duration (intensity: *p* < 0.001, frequency: *p* = 0.033, duration: *p* = 0.048)
	Thick	y = 0.578 + 0.613 × intensity − 0.155 × frequency + 0.078 × duration (intensity: *p* < 0.001, frequency: *p* = 0.005, duration: *p* = 0.013)
	Light	y = 2.088 − 0.427 × intensity + 0.12 × frequency − 0.078 × duration (intensity: *p* < 0.001, frequency: *p* = 0.044, duration: *p* = 0.023)
Two variables	Deep	y = 0.625 + 0.213 × intensity + 0.062 × duration (intensity: *p* < 0.001, duration: *p* = 0.006)
	Clear	y = 0.963 × intensity + 0.273 × duration (intensity: *p* < 0.001, duration: *p* < 0.001)
	Vibrating	y = 0.587 × intensity + 0.624 × duration (intensity: *p* < 0.001, duration: *p* < 0.001)
	Dense	y = 0.616 + 0.177 × intensity + 0.108 × duration (intensity: *p* = 0.008, duration: *p* < 0.001)
	Numb	y = 0.778 + 0.160 × intensity + 0.067 × duration (intensity: *p* = 0.032, duration: *p* = 0.012)
	Stubby	y = 1.109 + 0.397 × intensity − 0.125 × frequency (intensity: *p* < 0.001, frequency: *p* = 0.014)
	Shallow	y = 2.225 − 0.4 × intensity − 0.07 × duration (intensity: *p* < 0.001, duration: *p* = 0.038)
	Fuzzy	y = 2.330 − 0.327 × intensity − 0.142 × duration (intensity: *p* < 0.001, duration: *p* < 0.001)
	Soft	y = 1.433 − 0.320 × intensity + 0.106 × duration (intensity: *p* < 0.001, duration: *p* < 0.001)
One variable	Fast	y = 1.310 + 0.363 × intensity (*p* = 0.006)
	Pungent	y = 0.821 + 0.113 × intensity (*p* = 0.003)
	Skinny	y = 1.617 − 0.333 × intensity (*p* < 0.001)
	Thin	y =1.973 − 0.333 × intensity (*p* < 0.001)
	Slow	y = 1.053 + 0.064 × duration (*p* < 0.001)
	Ticklish	y = 0.943 + 0.128 × duration (*p* < 0.001)
	Tingling	y = 0.910 + 0.068 × duration (*p* = 0.001)
	Prickling	y = 0.688 + 0.078 × duration (*p* < 0.001)
	Tap	y = 2.321 − 0.313 × duration (*p* < 0.001)
	Rugged	y = 1.142 − 0.09 × frequency (*p* = 0.034)

**Table 2:** Polarity of Variables (Intensity, Frequency, Stimulation Duration) Affecting the Cognitive Characteristics.

Label	Constant	Intensity	Frequency	Stimulation Duration
Heavy	0.761	+	−	+
Bold	0.664	+	−	+
Thick	0.578	+	−	+
Light	2.088	−	+	−
Deep	0.625	+		+
Clear		+		+
Vibrating		+		+
Dense	0.616	+		+
Numb	0.778	+		+
Stubby	1.109	+	−	
Shallow	2.225	−		−
Fuzzy	2.330	−		−
Soft	1.433	−		+
Fast	1.310	+		
Pungent	0.821	+		
Skinny	1.617	−		
Thin	1.973	−		
Slow	1.053			+
Ticklish	0.943			+
Tingling	0.910			+
Prickling	0.688			+
Tap	2.321			−
Rugged	1.142		−	

## Data Availability

The datasets generated during this study are available from the corresponding author on reasonable request.
